# Evaluating allied health students’ readiness for placement learning

**DOI:** 10.1186/s12909-023-04005-w

**Published:** 2023-01-28

**Authors:** Belinda Judd, Jennie Brentnall, Justin Newton Scanlan, Kate Thomson, Felicity Blackstock, Allison Mandrusiak, Lucy Chipchase, Anna Phillips, Sue McAllister

**Affiliations:** 1 Faculty of Medicine and Health, University of Sydney, Camperdown, Australia; 2 School of Health Sciences, Western Sydney University, Campelltown, Australia; 3 School of Health and Rehabilitation Sciences, The University of Queensland, Brisbane, Australia; 4 College of Nursing and Health Sciences, Flinders University, Adelaide, Australia; 5 UniSA Allied Health and Human Performance, University of South Australia, Adelaide, Australia

**Keywords:** Assessment, Health professional students, Work integrated learning, Readiness, Allied health

## Abstract

**Background:**

Experiential learning opportunities, such as work integrated learning placements, are often challenging for health professional students. It is therefore imperative that students are adequately prepared before engaging in placement learning. Operationalising ‘readiness for learning on placement’ as a construct, is necessary for providing quality student feedback and assessment.

**Methods:**

An integrative mixed methods approach was adopted for this study, utilising a survey to canvass the perspectives of academics, students, and placement educators around the construct of readiness to inform potential assessment items. An assessment tool measuring student readiness for placement was then developed. Data from occupational therapy, physiotherapy and speech pathology programs were evaluated using Rasch analysis to explore the unidimensionality of this construct.

**Results:**

The online survey was completed by 64 participants, confirming the importance and measurability of foundational skills integral to readiness for placement learning. These foundational skills were then reflected in a pilot 20-item tool covering domains of professional and learner behaviour, communication, information gathering skills and reasoning. The Rasch analysis of 359 pre-registration student assessments confirmed unidimensionality, suggesting that the skills and attributes (operationalised as assessment items) that are considered part of ‘readiness for placement’ are components of this construct. Together, these findings provide support that the items on this tool are relevant and representative of the skills and behaviours that indicate readiness for placement learning. Two items regarding documentation and appropriate professional dress demonstrated some lower importance scores and interpretation variance warranting further investigation.

**Conclusion:**

Through the exploration of the construct of readiness for placement learning, we have created and subsequently revised, an innovative assessment tool that measures novice students’ pre-placement capabilities. Further research is now needed to explore the psychometric properties of the tool.

## Introduction

Work integrated learning (WIL) placements are a major component of allied health student education, providing essential instruction in both profession-specific and interdisciplinary skills, and experience in workplace roles and contexts [1]. Herein termed ‘placements’, these experiential learning opportunities situate pre-registration students in settings such as hospitals, private clinics, community, and aged care settings, where they engage in patient-related learning under the guidance of placement educators. Placements are resource-intensive for universities and healthcare organisations who invest time and effort in ensuring quality learning experiences that support student development, and assessment of competency to practice. Facilitating greater preparedness for placement may also help minimize the negative impacts of student underperformance on stakeholders [2]. Placement educators have an additional task of scaffolding students’ learning while maintaining patient care and safety, and placements can be a source of stress for both students [3] and educators [4]. Therefore, it is imperative that students are adequately prepared before engaging in placement-based learning [5, 6].

Universities increasingly seek authentic pedagogies to effectively prepare novice students to maximise their learning on placements. Simulation-based education has been adopted as a learning modality to support students to practice applying classroom learning in an authentic placement context [7]. Many allied health programs, particularly in Australia, have employed simulation-based education strategies to create learning environments that replicate workplace environments and professional tasks (simulated placements) [8], contrasting with discrete scenario-based simulations for subcomponents of tasks e.g., specific clinical procedures [9, 10]. These simulated placement environments provide scaffolded and controlled opportunities for students to practice and demonstrate a wide range of integrated patient-related skills and behaviours required in the workplace. Simulation, therefore, provides an ideal medium to explore novice students’ readiness for learning on placement. This learner-centered approach enables experiential learning opportunities that closely resemble the demands of real placements, provided in a minimal risk environment with the ability to control complexity and uncertainty [11, 12].

Health professional education and research have predominantly focussed on behaviours that indicate student readiness to join the profession in entry-level practice [13, 14]. Research specifically investigating ‘readiness for placement learning’ has identified relevant characteristics and skills but is yet to fully operationalise these constructs to assist with identifying whether novice students are ready. Chipchase and colleagues [6] explored attributes of a placement-ready student with placement educators in occupational therapy, physiotherapy, and speech pathology. Their Delphi study identified agreement on 57 characteristics and six main themes, with greater value on foundational, non profession-specific skills and qualities, such as willingness and professionalism. Similar findings have been reported in other health professions. In occupational therapy, being *ready* meant being ethical, responsible, and having good time management [15], and in dietetics, broader professional skills were essential [1]. In each of these studies, professional, ethical and learner behaviours were integral to educators’ perceptions of a student ready to learn on placement. There appears to be a consensus that the foundational skills that students require for placement learning (as perceived by educators) include communication skills, knowledge and understanding, and professional and learner behaviours [5, 6].

### Operationalising readiness for placement

‘Readiness for placement learning’ predominantly consists of non profession-specific skills and behaviours [6]. Thus, there is the potential to develop a unique standardised tool, applicable across multiple health professions, that provides a common language assessment approach and benchmarking competencies of students’ readiness to transition to learning on placement. Assessment tools measuring student performance must be robust and rigorously developed to enable sound judgements about student performance and provide quality feedback [16, 17]. The first step, however, is to operationalise ‘readiness for placement learning’ as a construct to enact the potential for quality student feedback and assessment. A major barrier is that determining competency in many of the characteristics and skills identified is challenging [18, 19].

Pre-placement skills are foundational to the development of professional competency during placement, which in turn enables development of entry-level health professional practice competency. These skills build on each other, in an example of McGaghie’s [20] mastery learning, that can be facilitated through scaffolding of each step. Students move on to the next level of learning only when ready, once the foundational building blocks have been laid and consolidated. The risk of moving on prematurely may result in the ultimate goal of entry-level practice competency not being achieved, with the student being overwhelmed and their confidence and learner agency impacted. Therefore, focusing on the early step of ‘readiness for placement’ development, feedback, and assessment is a critical step in the learner’s journey, providing the key foundational building blocks for success as a future health professional. The educational design therefore around ‘readiness for placement’ programs, learning outcomes and assessment, needs to be robust and well considered.

Operationalising the construct of ‘placement readiness’ would articulate what placement readiness ‘looks like’ and assure educators and students that progress towards readiness can be evaluated. University educators would subsequently be better placed to design learning experiences that enable novice students to practice and demonstrate behaviours that exemplify their readiness for progression to placement experiences. Supporting students to be well-prepared to commence learning on placement has potential benefits for all stakeholders. Being ‘ready’ to commence placements could reduce the high levels of stress and anxiety reported by students [21], as well as the negative impacts of student underperformance on the student, university, placement educators and service recipients.

The aim of this study was to articulate and operationalise the construct of ‘placement readiness’ in a tool suited to assess the readiness for placement learning demonstrated by novice allied health students undertaking simulation-based placement preparation programs. This study addressed the following research questions:

1. Is there agreement between allied health professional students, academics, and placement educators on behaviours that indicate students’ readiness to learn on placement?
2. What evidence is there for a unidimensional construct of ‘placement readiness’ that is applicable across professions that these behaviours represent?
3. What is the threshold of ‘placement readiness’ at which allied health professional students are perceived to be ready for learning on placement?

## Methods

### Study design

This study employed an integrative mixed methods design [22] to consider multiple stakeholder perspectives in operationalising the construct of ‘readiness for placement’. Considering readiness for placement as a candidate for assessment, it was framed as a unidimensional construct to which a Rasch Measurement Model [23] could be applied. The study’s integrative design then allowed deep consideration of how well the behavioural items and ratings of the tool operationalised this construct, inclusive of both qualitative and quantitative data and methods [22].

The study progressed in two stages. First, identifying behavioural indicators of students’ readiness for placement that could be evaluated in the context of a simulation-based placement preparation program. Secondly, evaluating whether these items sample a unidimensional construct of students’ readiness for placement, and if educators could identify different levels of performance consistently via a rating scale.

The study was approved by The University of Sydney Human Research Ethics Committee (HREC) prior to the recruitment of participants (approval no. 2017/658). All methods were performed in accordance with the Declaration of Helsinki. Participants were recruited separately for the two stages. Informed consent was obtained from all participants.

### Stage 1: Identifying behavioural indicators

In this stage, behavioural indicators that might represent student readiness for placement were first developed by the research team with reference to prior studies on the topic and informed by profession-specific examples of assessments of student competence. Item candidates were attributes that educators valued highly as indicating preparedness for placement [1, 6, 7, 15]. Items were considered if they were behaviourally demonstrable, might be observed in comprehensive simulation-based education programs used for placement preparation, and are common across a sample of available placement competence assessments in occupational therapy, physiotherapy, and speech pathology [24–26]. These items were compiled in a draft ‘Readiness for Placement Evaluation’ (RPE) tool.

Perspectives on the behavioural indicators comprising readiness for placement were then collected via an online survey of three stakeholder groups: students, simulation educators, and academics experienced with simulation. Three parallel questionnaires were developed, one for each participant group. These were developed by three of the authors who have diverse expertise in placement-based education (including preparation for placement programs) and measurement (BJ, JB, KT), and revised by other authors who have expertise in the construct of placement readiness (LC, AM) and in the development of performance assessments (SM). The surveys were then pilot tested with a small sample of students to check utility for this participant group who were not represented among the developers.

The stakeholder questionnaires included three questions asking respondents to rate each of the behavioural indicators in the draft RPE (Table 1). The exact wording of each question was tailored to the respondent group. Each question included an opportunity for participants to provide further information through qualitative comments. Each participant group was also asked to provide relevant demographic information.

**Table 1:** Summary of stakeholder questionnaires

Topic	Response Format
Whether the behavioural indicator was demonstrable during the respondent’s preparation program	Yes | Not sure | No
The importance of the behavioural indicator in determining a student’s placement readiness	Seven-point Likert scale: 1 = not important to 7 = extremely important
The minimum level of performance required to demonstrate a student’s placement readiness	Five-point Likert scale: 0 = not demonstrated at appropriate opportunities 1 = rarely demonstrated without significant prompting and performed below acceptable standard 2 = demonstrated appropriately with some prompting and performed at an acceptable standard 3 = consistently demonstrated appropriately with minimal prompting and performed at an acceptable standard 4 = consistently demonstrated above expectations for placement readiness

A purposive sampling approach [27] was used to recruit participants from the three groups. The simulation-based educators and students were recruited through the degree programs included in this study at the host institution, and academics were recruited from around the country through a snowballing approach commencing with the authors’ professional networks. Educators and academics were eligible for inclusion if they had been involved in implementing a least one simulation-based placement preparation program in entirety. Students were eligible for inclusion if they were current students who had completed a simulation-based program in preparation for placement in the two years prior to the survey and had subsequently completed at least one placement.

The survey design, by seeking to obtain predominantly subjective and descriptive information, had a suggested sample size of approximately 40–60 participants [28–30]. We therefore aimed to recruit approximately 60 participants with equal representation across the three stakeholder groups. Email invitations containing a link to the online survey were used to recruit participants, with a reminder email sent 1–2 weeks after the initial email.

### Stage 2: Designing and piloting a tool to evaluate the utility of operationalising behavioural indicators

In this stage, we finalised, and pilot tested the Readiness for Placement Evaluation (RPE) to assess students’ development of placement readiness in profession-specific, simulation-based placement preparation programs for each of the three professions.

The RPE featured 20 items in four domains, with each item rated on a 5-point scale with a descriptor for each level. This design maintained familiarity for raters, being common to other widely adopted student assessments [24, 25]. At the end of the assessment, the placement educator completed a summary global rating of the students’ readiness for placement. The four-point summary rating scale recorded educators’ judgement whether students were not ready to progress, recommended to progress after further specific remediation, ready to progress with no further action, or performing above expectations.

The simulation-based placement programs for each profession took place in a purpose-built clinical simulation centre on the grounds of a large, metropolitan university. The centre comprised a six-bed acute care ward with bathroom, inpatient rehabilitation gym, and an outpatient treatment area with adjacent modified bathroom and kitchen and a separate patient waiting area. In each program, simulated patients were played by professional actors trained to portray clinical cases based on deidentified real patient cases. Simulated patients were the modality best suited to the learning outcomes focussing on novice professional communication and other generic foundational skills. Simulated patients were adorned with authentic attachments relevant to their condition such as drips, drains, and limb braces. Associated indirect patient-related activities were also incorporated into programs to further replicate aspects of authentic work placements in a manner that facilitated the development of foundational skills. These included facilitating students’ preparations for the placement tasks of patient file review, patient interviewing, physical examination, simple patient treatments or recommendations, progress note documentation, referrals, and patient handovers/discharge planning. The programs ran intensively across one week for physiotherapy and speech pathology students and as five, sequentially linked, one-day sessions integrated throughout a semester-long module for occupational therapy students. Each program was repeated as frequently as needed in each teaching period to accommodate the numbers of students enrolled in each program.

The RPE assessment was completed for each student by their supervising educator at the conclusion of their program. To promote consistency, information on the RPE and expected standards were incorporated in the educator (and student) briefing sessions, and each educator was provided with overall scoring guidelines and example behavioural descriptors for each item. De-identified RPE data were obtained from each unit of study coordinator after students were provided an opportunity to opt out. This approach was used so data sets would not be subject to influence by patterns of student volunteers, and without additional participant information that may contribute to bias.

#### Data analysis

Data analysis in both stages (survey and assessment tool data) integrated qualitative and quantitative components to explore the measurement of students’ readiness for placement with respect to the research questions.

Survey responses were analysed descriptively, examining the similarities and differences in responses across the participant groups [31]. For the question regarding the relative importance of each of the behavioural indicators in determining a students’ placement readiness, the 7-point Likert scale was collapsed to three levels: low importance (score of 1–2), moderate importance (score of 3–5), or high importance (score of 6–7). All participant free text comments were collated and analysed descriptively by one author (KT) and checked for consistency and interpretability by a second author (BJ). These comments aided the overall interpretation of results.

For the assessment tool data, Rasch analysis (using Winsteps software version 4.0.1) was adopted as a robust approach to investigate how well the items and process of the RPE represented placement readiness. Rasch analysis has frequently been adopted in the development and investigation of placement performance assessments in allied health [32–35]. Rasch analysis is an appropriate, well-published method to investigate unidimensionality, and thereby coherence, of a construct represented in a tool, and thus to explore whether the operationalised items reflect the construct of readiness for placement. The Rasch approach also considers whether different levels of performance can be identified using the tool [23].

The completeness of data was investigated to illustrate the observability of the items and feasibility of completion. Rasch analyses of *fit* of data to the assumptions of the Rasch Measurement Model (correlations between item and total scores and mean square fit statistics) were used to examine the unidimensionality of the construct represented by the behavioural indicators [23]. Further analyses investigated the use of the rating scale categories in these programs, and the ability of the ratings to distinguish statistically distinct levels of performance. The relative difficulties of behavioural items and thresholds of scoring representing perceived readiness were investigated, including with reference to qualitative indicators and the Stage 1 data to confirm the representation of the construct of interest. The extent to which assumptions of the Rasch Measurement Model held across professions was investigated in tests of differential item functioning (DIF) [36]. This data analysis was carried out by one author (JS) and checked for consistency and interpretability by two other authors (BJ and JB).

## Results

### Participants

*Stage 1*: The survey participants (*n =* 64) represented the three target groups with representatives from different professions (Table 2.). The students were of different degree levels (*n =* 19 Master; *n =* 17 Bachelor), and the placement educators ranged from inexperienced (supervised less than 10 students) to those who had hosted over 100 students across multiple years.

**Table 2:** Survey participant characteristics

	Academics	Simulation Educators	Students
Occupational Therapy	3	6	9
Physiotherapy	8	5	9
Speech Pathology	4	2	18
Total (*n =* 64)	15	13	36

*Stage 2*: The 359 RPE student evaluations were collected in simulation-based placement preparation programs and no students opted out of their inclusion. Included were evaluations by placement educators of one cohort each of physiotherapy (*n =* 233), occupational therapy (*n =* 78) and speech pathology (*n =* 48) students.

The results are presented as an integrated overview from the combination of survey data and Rasch analysis addressing four components; the three research questions, followed by the overall outcome of the study.

#### Agreement between academics, placement educators and students on behaviours that indicate readiness to learn on placement

A total of 20 behavioural indicators were developed, sampling multiple subthemes under each of four key domains, whilst maintaining useability of the assessment format. The four domains covered were; professional behaviour, learner behaviour, communication and information gathering, and the proposed instrument was named the *Readiness for Placement Evaluation (RPE)*.

First, survey participants were asked if a student could demonstrate each of the 20 behavioural items during a simulation-based placement. Seventy percent (*n =* 45/64) agreed that all 20 of the behavioural items listed were able to be demonstrated in their simulation-based preparation programs. Of the remaining 30% of respondents, the three items that attracted the largest response of ‘*Not sure*’ or ‘*No*’ were relatively consistent in being identified by at least two of the three participant groups. These were ‘produces clear and accurate documentation’ (Item 3.3; simulation educator and student groups reported less agreement that this was demonstrable), ‘contributes to patient/client-centred goal setting and recommendations (Item 4.5; reported by all three groups), and ‘contributes to workplace routines and operates effectively as a team member’ (Item 1.4; reported by the student and academics groups) see Table 3.

**Table 3:** Summary of main RPE assessment tool item findings

Competency	Consistent Demonstrability	Importance	Threshold for readiness
**Professional Behaviour**			
1.1 adheres to professional and ethical standards including privacy, informed consent, and confidentiality	✓	✓	Perceived higher by educators and academics than students
1.2 is punctual and manages their application of time to tasks	✓	✓	✓
1.3 presents with appropriate professional dress and appearance^a^	Lower fit of item observations	Perceived lower by all groups	Measured as easier for students’ item
1.4 contributes to workplace routines and operates effectively as a team member	Less agreement to demonstrability by students and academics	✓	✓
1.5 identifies and responds to potential risks and hazards	Less frequently observed	✓	✓
**Learner Behaviour**			
2.1 shows initiative and willingness to learn	✓	✓	Perceived higher by all groups
2.2 takes responsibility for their own learning	✓	✓	Perceived higher by all groups
2.3 demonstrates awareness of own limitations	✓	✓	✓
2.4 seeks and responds appropriately to feedback	✓	Perceived highest of all items	✓
2.5 demonstrates organisational and problem-solving skills	✓	✓	Measured more difficult for these students
**Communication**			
3.1 communicates professionally with peers and staff	✓	✓	✓
3.2 demonstrates effective communication and interpersonal skills with patient/client	Lower fit of item observations	✓	✓
3.3 produces clear and accurate documentation^a^	Less agreement to demonstrability by educators and students; Less frequently observed	Perceived lower by all groups	Perceived lower; Measured more difficult for these students
3.4 demonstrates sensitivity and empathy to patient/client needs and concerns	Lower fit of item observations	✓	✓
3.5 communicates effectively in a team setting	✓	✓	✓
**Information Gathering**			
4.1 plans and prepares relevant and appropriate information gathering processes	✓	✓	✓
4.2 implements information gathering strategies effectively including file review and history taking	✓	✓	✓
4.3 identifies important and relevant patient/client information	✓	✓	Perceived lower by all groups
4.4 interprets information to identify patient/client main problems	✓	✓	Perceived lower; Measured more difficult for these students
4.5 contributes to patient/client-centred goal setting and recommendations	Less agreement to demonstrability by all groups	✓	✓

^a^item requires further investigation

The second survey question addressed the relative importance of each of the behaviours (20 items) in indicating whether a student is ready to learn in a placement setting. All three stakeholder groups rated all items as either moderate or high importance, with student respondents showing less differentiation of importance between items than the academic and placement educator respondents. The items ranked lower in importance (‘moderate’) across all groups were ‘produces clear and accurate documentation’ (Item 3.3), and ‘presents with appropriate professional dress and appearance’ (Item 1.3). The item ‘seeks and responds appropriately to feedback’ (Item 2.4) was the most highly scored item for importance.

From the pilot assessment tool data, only two of the 20 items had more than 10% missing data, signalling that educators did not always observe these two items in the simulation preparation program in order to score them. These items were ‘identifies and responds to potential risks and hazards’ (Item 1.5), and ‘produces clear and accurate documentation’ (Item 3.3), which were not rated 24% and 48% of the time respectively.

#### Evaluating the utility of operationalising behavioural indicators; evidence of a unidimensional construct of placement readiness

From the pilot assessment tool data, correlations between item scores and total scores were positive for all items and most items had infit and outfit mean square statistics within the acceptable range (MnSq 0.6 to 1.4), indicating adequate fit to the expectations of the Rasch Measurement Model. Three items had fit statistics that fell just outside of this range (MnSq 1.47–1.55): ‘presents with appropriate professional dress and appearance’ (Item 1.3); ‘demonstrates effective communication and interpersonal skills with patients/clients’ (Item 3.2) and ‘demonstrates sensitivity and empathy to patient/client needs and concerns’ (Item 3.4). These results indicate slightly larger variation in item scoring and interpretation, or less predictable judgement of student performance on these specific RPE items.

Each higher rating category on the RPE scale had a higher average measure than the previous in a monotonic progression, with all outfit mean squares < 2.0 logits. Categories on the lower end of the scale were relatively under-utilised, and the upper end of the scale more heavily utilised. Even so, each step difficulty progressed satisfactorily by > 1.4 logits, although the final step progression was larger than 5.0, suggesting that raters perceive a wide difference in student ability between the second highest (3) and highest (4) categories on the scale. Despite the heavy use of the upper scores on the 5-point rating scale, less than 3% of students in the pilot study attained a full score on every item (ten student assessments out of a total of 359). The person separation index was 4.00 and the person separation reliability was 0.94. That is, four levels of student readiness for placement could be distinguished using this assessment for this student population.

**Fig. 1:**
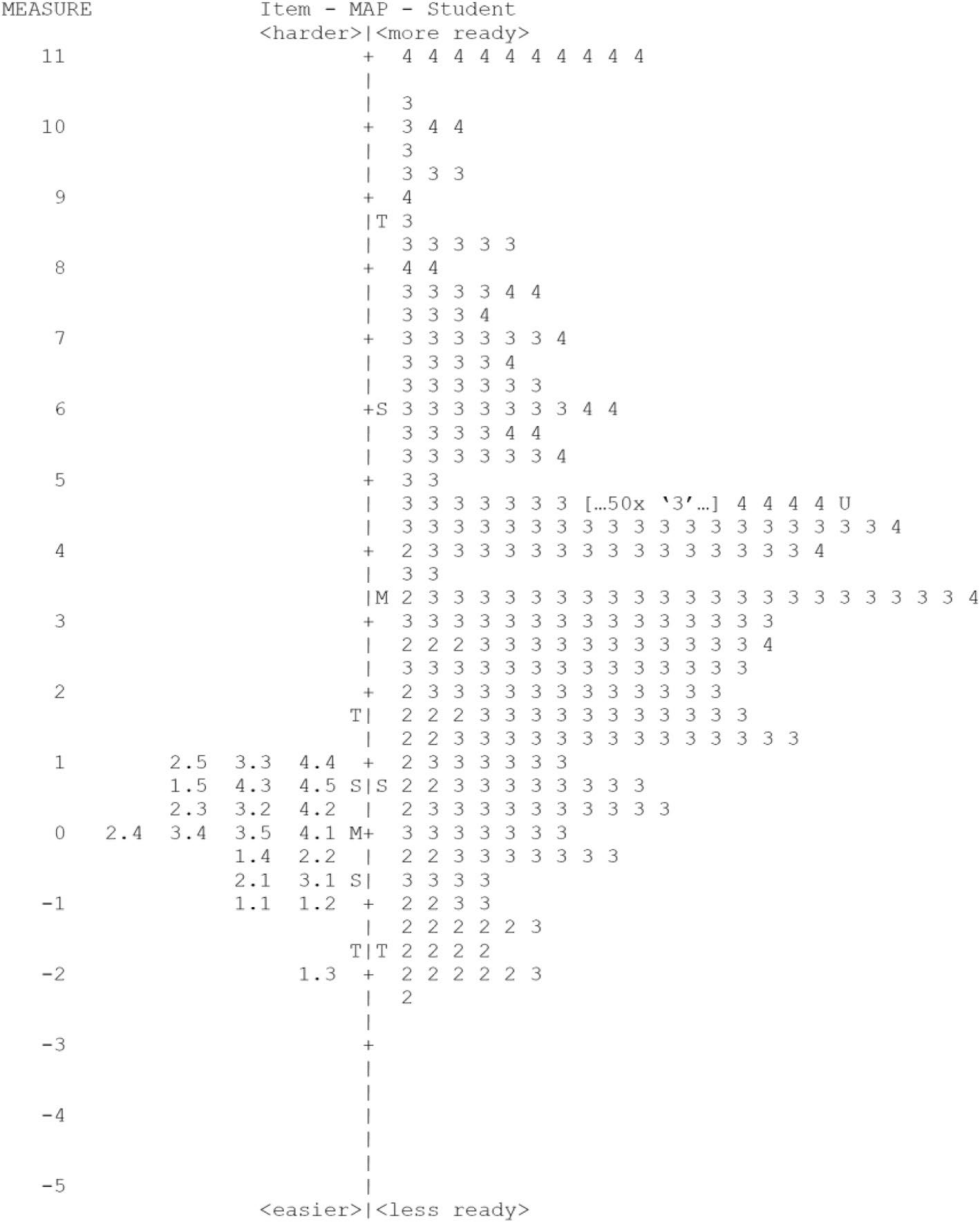
Item-student map Item-student map representing all assessment data. Item difficulty (left of centre line) are mapped against student abilities (right of centre line). Students' ability is arranged from highest performing to lowest performing, and item difficulty is from most difficult to least difficult. The mean student ability ('M' on right of centre line) is higher than the mean of item difficulty ('M' on left of centre line). S = 1 standard deviation, T = 2 standard deviations

Examination of the student-item map (Fig. 1.) reveals that most items are clustered around the bottom of the map which aligns with this propensity toward use of the upper end of the scale (high scores) and a wide range between upper rating categories on the scale. Results indicate that raters were highly likely to rate students no lower than the mid-point rating on most items. The Rasch measure score was also moderately correlated to the global rating scale of readiness at the conclusion of the evaluation form (Spearman 0.525, *p* < 0.001).

There was some evidence of differential item functioning (DIF; having different probabilities of obtaining the same score on a given item after controlling for other item observations) between occupational therapy and physiotherapy student evaluations that warrants further examination of two items: ‘identifies and responds to potential risks and hazards’ (Item 1.5) and ‘produces clear and accurate documentation’ (Item 3.3). These two items may be interpreted somewhat differently by educators in these different professions, noting that these two items were also the items most frequently not scored.

#### Evaluating the utility of operationalising behavioural indicators; Thresholds of performance indicating students are ready to begin clinical placement

The final survey question explored the standard of performance for each item that the participants perceive demonstrates readiness for learning on placement. There was high consistency across the three groups, suggesting that students, placement educators and academics all agree on what support is appropriate for a placement-ready student. On the 0–4 scale, higher ratings were reported to represent the threshold of placement readiness for items such as ‘shows initiative and willingness to learn’ (Item 2.1), ‘takes responsibility for their own learning’ (Item 2.2), and ‘presents with appropriate professional dress and appearance’ (Item 1.3), indicating that students who are ready for placement should be able to demonstrate these behaviours without prompting. Lower ratings, indicating more prompting and support prior to placement, were made for items requiring higher level clinical reasoning. These were, ‘identifies important and relevant patient/client information’ (Item 4.3), ‘interprets information to identify patient/client main problems’ (Item 4.4), and ‘produces clear and accurate documentation’ (Item 3.3). One behavioural item that differed in performance expectation across the groups was ‘adheres to professional and ethical standards including privacy, informed consent and confidentiality’ (Item 1.1), where less prompting was expected to demonstrate ‘readiness’ to simulation educators and academics as compared to students.

Overall, the Rasch analysis results from the pilot assessment tool data are consistent with the survey findings and demonstrated a stable and predictable item hierarchy, with items concerning professional and learner behaviours being the easiest to score highly on, and items requiring reasoning skills and interpretation of information being the most difficult items to score highly on. Rasch analysis also demonstrated that items are clustered in terms of difficulty around the crucial transition point on the global rating scale between categories labelled ‘*Requires remediation prior to Clinical Placement*’ (category 2) and ‘*Is ready for Clinical Placement*’ (category 3). Having several items allows greater measurement precisions at this crucial point of decision-making, about readiness to learn on placement (Fig. 1).

#### Outcome: Evolution of the Readiness for Placement Evaluation (RPE) into the Evaluation of Foundational Placement Competencies (EFPC)

Following all data analyses, minor revisions were made to the RPE to create the *Evaluation of Foundational Placement Competencies* (EFPC items). The four-domain structure was retained, with minor changes to wording of some items or their corresponding performance descriptors based on the study results. For example, an extra explanatory sentence was added to the performance descriptor of a communication item for enhanced clarity. The summary rating scale was revised to more closely reflect educator overall recommendations of the extent to which students met foundational standards. In the resulting 20-item tool, there is good evidence for retaining 18 of the items. The two items concerning ‘documentation’ and ‘appropriate dress’, require further investigation to establish if these skills and behaviours are part of the construct of ‘student readiness for placement’ across professions *and* are feasible to evaluate in a range of placement preparation program designs.

## Discussion

This study operationalised the construct of placement readiness in an evaluation of novice students’ demonstrated behaviours in simulation-based placement preparation programs. A survey of 64 academics, simulation educators, and students confirmed the representation of the construct of placement readiness in the 20 items, regarding most to be measurable in pre-placement simulation programs and demonstrating high consistency between the stakeholder groups. Rasch analysis of 359 student assessments from three health professions confirmed the utility and suitability of the proposed tool and that it measured a single construct of placement readiness. The items around documentation and appropriate dress/uniform may require further investigation.

The operationalisation of placement readiness into a tool measuring novice allied health student readiness for learning in placement contexts provides academics, placement educators and students with guidance regarding behavioural expectations of students. The high consistency of survey responses confirmed that the items (competencies) represent behaviours indicative of and necessary for placement readiness and that can be demonstrated in simulation-based placement preparation programs. The alignment between participant perceptions and the literature accords with the basis of the tool in prior research on student readiness for placement learning [5, 6, 15]. This current study adds weight to that literature and newly integrates educator perspectives with that of students and academics.

A characteristic of this pre-placement student evaluation is that student performance is rated as demonstrated throughout an experience during which there are multiple opportunities where the student could demonstrate each behaviour. In this study, these opportunities were provided in 5-day simulation programs with standardised patients. This approach to gathering more longitudinal evidence regarding a students’ placement readiness is similar to that used to assess the performance of allied health students in clinical placements [25, 26, 33, 37]. This provides familiarity for the educators rating students, easing the cognitive burden involved in assessing multiple students, and promotes acceptance of the relevance of the tool. Further, this approach accords with contemporary learning theory and programmatic assessment models which highlight the need for basing judgements of performance on multiple samples of behaviour [38] rather than a high-stakes assessment at a single point in time. Avoiding a single, high-stakes, moment of assessment and providing opportunity for a developmental approach where students can learn from experiences, integrate feedback, and improve performance also supports efforts to maintain psychological safety for learners during simulation-based education [39].

To ensure a clear and feasible decision regarding readiness for placement across students and settings, further exploration of the items on ‘professional dress’ and ‘documentation’ is warranted. Survey respondents in this study considered those competencies to be less relevant to evaluating students’ readiness for placement, yet placement educators have previously rated appropriate professional dress and appearance as very important and written communication including in charts as important [6]. It is not clear if the lower importance placed on these items reflects the campus-based nature of simulation-based preparation programs and particularly the lack of interactions with a health professional team through documentation, perceived variances in requirements between different placement settings, or other factors. The ‘professional dress’ item also showed lower fit between the observed ratings and those predicted by the Rasch model, suggesting it is not necessarily aligned with the development of ‘readiness for placement’. While the observed ratings for documentation were better aligned with the Rasch model expectations in this study, this was less frequently rated. A study adapting a speech pathology placement assessment to evaluate pre-novice competencies in a simulation program similarly found that educators’ ratings of students were less predictable for several elements of the professionalism competency, including appearance and dress, the ‘other written records’ element of the communication competency and a lifelong learning element with no direct equivalent in this study [37]. In other studies, applying the Rasch measurement model, documentation has similarly proved problematic to measure in physiotherapy entry-level assessments [32], and the widely used entry-level occupational therapy assessment has shown multidimensionality [35].

In incorporating student views, this study aimed to base the tool on stakeholder consensus on agreed expectations. It is notable that students tended to rate all competencies as having similar and high importance. While the academics and simulation educators rated all items as having moderate or high importance, the even more constrained range of ratings by students suggests that they may have difficulty differentiating these competencies or a less nuanced understanding of placement readiness. For most students, this is of limited consequence since they can meet all the expectations. However, if students are unclear on priorities when listening to constructive feedback, this may impact on their development of placement readiness. Further, the student cohorts surveyed had already had placement experience and may therefore have more developed understanding of the contextual standards and performance and of evaluative judgement [40] than the intended audience for this tool. These findings suggest that when working with less experienced students, such as those undertaking pre-placement preparation programs, outlining learning outcomes and assessment criteria clearly at the start of the experience may need further emphasis and explicit instruction during program briefing. Use of this tool and the results of this research on the hierarchy of difficulty of the items may provide students and educators alike with a clear method of detailing the measurable endpoint of the pre-placement program.

According with the selection of behaviours considered important for screening students, the results demonstrated that the assessment items were generally easy to score highly on relative to student ability. This creates a possible “ceiling effect” in the tool, with reduced measurement precision for distinguishing between students with higher levels of ability [23]. At this end of the scale, the 5-point rating scale for each item resulted in less than 3% of students in the pilot study attaining a full score despite the propensity to score highly overall. This ceiling effect is not problematic given the tools’ purpose. The peak measurement precision of the tool aligns well with the purposes of evaluating placement readiness, distinguishing between those students who are ready to progress onto subsequent clinical placements and those who may need further development or are clearly not ready.

Behaviours thought to demonstrate placement readiness are largely uniform across professions [6], and this was confirmed by performance ratings that were consistent across three allied health professions and varied simulation program designs within one institution. The application of a standard tool across the professions allows streamlining of processes, supports interprofessional learning programs, and facilitates benchmarking of both student performance and preparation programs. The low standard expected for the two items; ‘the student produces clear and accurate documentation’ and ‘contributes to patient/client-centred goal setting and recommendations’ and high standard for ‘the student shows initiative and willingness to learn’ may suggest that the construct of readiness is more about students’ approach to learning rather than specific skills related to disciplinary practice. Additionally, we only observed minor differential item functioning (DIF) for two items across the three professions (‘identifies and responds to potential risks and hazards’; Item 1.5 and ‘produces clear and accurate documentation’; Item 3.3). Educators from different professions typically interpreted the meaning of items comparably. This aligns with findings in previous research [1, 6, 15] and provides evidence for the focus on development of positive learner behaviours in preparation for placement programs. The results of this study can be used to guide students in their learning, academics in their curriculum design, and placement educators in their scaffolding strategies The tool can be applied to define standards and inform learning, including in formative assessment for learning as students approach the time when placement readiness is required. Educators should consider focusing on developing students’ professional behaviours, learner attributes, and communication and information gathering, when designing placement preparation programs. This is considering the consistently high ratings of importance to commencing placement of each of these behaviours. Given these skills and attitudes do not appear to be profession-specific and therefore preparation programs could incorporate interprofessional learning whilst adopting a common assessment tool that has included the student voice in its development.

### Strengths, limitations, and recommendations for future research

The exploration of the construct of readiness for placement and subsequent tool development included multiple stakeholder perspectives and was trialled across three allied health professions. However, the specific assessment data collected was from a single institution. Broader exploration of validity of score interpretations would further add to the robustness of the tool. This should focus on ensuring generalisability across different curricula, and how student evaluations are reflected in readiness for subsequent placements with or without recommended further development. Further research may also include other health professions with similar placement models and expectations of placement readiness. Exploration of the construct of readiness for work integrated learning in non-health professions such as business where placement models more typically include internships and industry projects is another area that warrants further research.

The survey sampling strategy included snowballing, which likely attracted highly interested and networked participants that may not be representative of the three target groups. It also results in not being able to ascertain a true response rate. However, those data were not used alone as representative of the perspectives of placement educators, academics, and students. Rather, those data served a useful role to complement and extend both existing literature on preparing students for placement and the Rasch analysis of the tool.

Further research could also explore the suitability of the tool for student self and peer assessment. This warrants particular consideration given the differences between groups apparent in this study. A tool suited to self and peer assessment would help students to identify the competencies they need to focus on developing and demonstrating during their preparation program, and those competencies that they already have developed. This knowledge would assist students to maximise the benefits of preparation time and increase their understanding around placement expectations. It is, however, contingent on the characteristics of the tool whilst potentially also contributing to developing evaluative judgement through opportunities to evaluate their own and peers’ placement readiness and to reflect critically on their performance in placement preparation programs [41].

## Conclusion

This study explored the construct of readiness for placement learning and found that there was agreement by students, academics, and placement educators on 20 items that described behaviours across four categories: professional behaviour; learner behaviour; communication; and information gathering. Rasch analysis confirmed that these items sampled a unidimensional construct of ‘placement readiness’ that is applicable across three diverse allied health professions and that threshold behaviours indicative of ‘placement readiness’ had been identified. Further empirical research is needed to explore the validity of judgements made using the Evaluation of Foundational Placement Competencies (EFPC) tool, including over time and in placement preparation programs at other institutions. However, the EFPC shows promise for supporting student learning, including interdisciplinary learning activities, and promoting positive outcomes for all stakeholders by identifying students who are likely to be successful on their first placement.

## Data Availability

The datasets used and/or analysed during the current study are available from the corresponding author on reasonable request.
